# A rule-based ontological framework for the classification of molecules

**DOI:** 10.1186/2041-1480-5-17

**Published:** 2014-04-15

**Authors:** Despoina Magka, Markus Krötzsch, Ian Horrocks

**Affiliations:** 1 Department of Computer Science, University of Oxford, Oxford, UK; 2 Department of Computer Science, Technical University of Dresden, Dresden, Germany

**Keywords:** Semantic technologies, Knowledge representation and reasoning, Logic programming and answer set programming, Datalog extensions, Cheminformatics

## Abstract

**Background:**

A variety of key activities within life sciences research involves integrating and intelligently managing large amounts of biochemical information. Semantic technologies provide an intuitive way to organise and sift through these rapidly growing datasets via the design and maintenance of ontology-supported knowledge bases. To this end, OWL—a W3C standard declarative language— has been extensively used in the deployment of biochemical ontologies that can be conveniently organised using the classification facilities of OWL-based tools. One of the most established ontologies for the chemical domain is ChEBI, an open-access dictionary of molecular entities that supplies high quality annotation and taxonomical information for biologically relevant compounds. However, ChEBI is being manually expanded which hinders its potential to grow due to the limited availability of human resources.

**Results:**

In this work, we describe a prototype that performs automatic classification of chemical compounds. The software we present implements a sound and complete reasoning procedure of a formalism that extends datalog and builds upon an off-the-shelf deductive database system. We capture a wide range of chemical classes that are not expressible with OWL-based formalisms such as cyclic molecules, saturated molecules and alkanes. Furthermore, we describe a surface ‘less-logician-like’ syntax that allows application experts to create ontological descriptions of complex biochemical objects without prior knowledge of logic. In terms of performance, a noticeable improvement is observed in comparison with previous approaches. Our evaluation has discovered subsumptions that are missing from the manually curated ChEBI ontology as well as discrepancies with respect to existing subclass relations. We illustrate thus the potential of an ontology language suitable for the life sciences domain that exhibits a favourable balance between expressive power and practical feasibility.

**Conclusions:**

Our proposed methodology can form the basis of an ontology-mediated application to assist biocurators in the production of complete and error-free taxonomies. Moreover, such a tool could contribute to a more rapid development of the ChEBI ontology and to the efforts of the ChEBI team to make annotated chemical datasets available to the public. From a modelling point of view, our approach could stimulate the adoption of a different and expressive reasoning paradigm based on rules for which state-of-the-art and highly optimised reasoners are available; it could thus pave the way for the representation of a broader spectrum of life sciences and biomedical knowledge.

## Background

Life sciences data generated by research laboratories worldwide is increasing at an astonishing rate turning the need to adequately catalogue, represent and index the rapidly accumulating bioinformatics resources into a pressing challenge. Semantic technologies have achieved significant progress towards the federation of biochemical information via the definition and use of domain vocabularies with formal semantics, also known as *ontologies*[1-3]. OWL [4], a family of logic-based knowledge representation (KR) formalisms standardised by the W3C, has played a pivotal role in the advent of Semantic technologies. This is to a great extent thanks to the availability of robust OWL-based tools that are capable of deriving knowledge that is not explicitly stated by means of logical inference. In particular, OWL bio- and chemo-ontologies with their intuitive hierarchical structure and their formal semantics are widely used for the building of life sciences terminologies [5,6].

Taxonomies provide a compelling way of aggregating information, as hierarchically organised knowledge is more accessible to humans. This is evidenced, e.g. by the pervasive use of the periodic table in chemistry, one of the longest-standing and most widely adopted classification schemes in natural sciences. Organising a large number of different objects into meaningful groups facilitates the discovery of significant properties pertaining to that group; these discoveries can then be used to predict features of subsequently detected members of the group. For instance, esters with low molecular weight tend to be more volatile and, so, a newly found ester with low weight is expected to be highly volatile, too. As a consequence, classifying objects on the basis of shared characteristics is a central task in areas such as biology and chemistry with a long tradition of taxonomy use. Due to the availability of performant OWL reasoners, life scientists can employ OWL to represent expert human knowledge and thus drive fast, automatic and repeatable classification processes that produce high quality hierarchies [7,8]. Nevertheless, a prerequisite is that OWL is expressive enough to model the entities that need to be classified as well as the properties of the superclasses that lie higher up in the hierarchy.

Two main restrictions have been identified in the expressive power of OWL as hindering factors for the representation of biological knowledge [9,10]. First, due to the tree-model property of OWL [11] (which otherwise accounts for the robust computational properties of the language) one is not able to describe cyclic structures with adequate precision. Second, because of the open-world assumption adopted in OWL (according to which missing information is treated as *not known* rather than *false*) it is difficult to define classes based on the absence of certain characteristics. These limitations manifest themselves—among others—via the inability to define a broad range of classes in the chemical domain. For instance, one cannot effectively encode in OWL the class of compounds that contain a benzene ring or the class of molecules that do not contain carbon atoms, i.e. inorganic molecules.

These inadequacies obstruct the full automation of the classification process for chemical ontologies, such as the ChEBI (**Ch**emical **E**ntities of **B**iological **I**nterest) ontology, an open-access dictionary of molecular entities that provides high quality annotation and taxonomical information for chemical compounds [6]. ChEBI fosters interoperability between researchers by acting as the primary chemical annotation resource for various biological databases such as BioModels [12], Reactome [13] and the Gene Ontology [5]. Moreover, ChEBI supports numerous tasks of biochemical knowledge discovery such as the study of metabolic networks, identification of disease pathways and pharmaceutical design [14,15]. ChEBI is manually curated by human experts who annotate and check the validity of existing and new molecular entries. Currently, ChEBI describes 36,660 fully annotated entities (release 110) and grows at a rate of approximately 4,500 entities per year (estimate based on previous releases [16]). Given the size of other publicly available chemical databases, such as PubChem [17] that contains records for 19 million molecules, there is clearly a strong potential for ChEBI to expand by speeding up curating tasks. ChEBI curating tasks span a wide range of activities such as adding natural language definitions and structure information or classifying chemical entities by determining their position in the ChEBI taxonomy. Thus automating chemical classification could free up human resources and accelerate the addition of new entries to ChEBI.

As the classification of compounds is a key task of the drug development process [18], the construction of chemical hierarchies has been the topic of various investigations capitalising on logic-based KR [19-23], statistical machine learning (ML) [24-26] and algorithmic [27-29] techniques. In KR approaches, molecule and class descriptions are represented with logical axioms crafted by experts and subsumptions are identified with the help of automated reasoning algorithms; in ML approaches a set of annotated data is used to train a system and the system is then employed to classify new entries. So, KR approaches are based on the explicit axiomatisation of knowledge, whereas ML algorithms specify for new entries superclasses that are highly probable to be correct. As a consequence, the taxonomies produced using logic-based techniques are provably correct (as long as the modelling of the domain knowledge is faithful), but the statistically produced hierarchies (although much faster) need to be evaluated against a curated gold standard. Algorithmic techniques involve the definition of imperative procedures for determining classes of molecules. These approaches are usually much quicker than logic-based techniques but have the disadvantage of requiring a programmer for defining new classes or for modifying the existing ones, as opposed to ontological knowledge bases that can be manipulated and extended by non-programmers. Here, we focus on logic-based chemical classification, which in certain cases can complement statistical and algorithmic approaches [8,15].

In previous work, we laid the theoretical foundation of *nonmonotonic existential rules* which is an expressive ontology language that is sound and complete and that is suitable for the representation of graph-shaped objects; additionally, we demonstrated how nonmonotonic existential rules can be applied to the classification of molecules [9]. The aforementioned formalism addressed the expressivity limitations outlined above; however, the performance of the implementation—although faster than previous approaches—was not satisfactory (more than 7 minutes were needed to classify 70 molecules under 5 chemical classes on a standard desktop computer) failing thus to confirm practicability of the formalism.

In the current work, we describe an improved practical framework that relies on the same formalism but with enhanced performance. Our contributions can be summarised as follows: 

1. We present a prototype that performs logic-based chemical classification based on a sound, complete and terminating reasoning algorithm; we model more than 50 chemical classes and we show that the superclasses of 500 molecules are computed in 33 seconds.

2. We harness the expressive power of nonmonotonic existential rules to axiomatise a variety of chemical classes such as classes based on the containment of functional groups (e.g. esters) and on the exact cardinality of parts (e.g. dicarboxylic acids), classes depending on the overall atomic constitution (e.g. hydrocarbons) and cyclicity-related classes (e.g. compounds containing a cycle of arbitrary length or alkanes).

3. We present a *surface syntax* that enables application experts to create ontological description of chemical entities without prior knowledge of logic. The syntax we propose is closer to natural language than to first-order logic notation and is uniquely translatable to logical axioms.

4. We exhibit a significant speedup in comparison with previous ontology-based chemical classification implementations.

5. We identify examples of missing and contradictory subsumptions from the expert curated ChEBI ontology that are present and absent, respectively, from the hierarchy computed by our prototype.

Concerning future benefits, our prototype could form the basis of an ontology-mediated application to assist biocurators of ChEBI towards the sanitisation and the enrichment of the existing chemical taxonomy. Automating the maintenance and expansion of ChEBI taxonomy could contribute to a more rapid development of the ChEBI ontology and to the efforts of the ChEBI team to make annotated chemical datasets available to the public. From a modelling point of view, our approach could stimulate the adoption of a different and expressive reasoning paradigm based on rules for which state-of-the-art and highly optimised reasoners are available; it could thus pave the way for the representation of a broader spectrum of life sciences knowledge.

## Methods

### Knowledge base design

The reasoning task carried out using our methodology is the identification of chemical classes for molecules, e.g. assigning water to the class of inorganic molecules or benzene to cyclic molecules. In this section we provide a high-level description of the knowledge base (KB) we built for the purposes of our chemical classification experiments. We use the word ‘classification’ to refer to the detection of subsumptions between molecules and chemical classes rather than to the computation of the partial order for the set comprising the chemical classes and molecules w.r.t. the subclass relation. The KB consists of nonmonotonic existential rules that formally describe molecular structures and chemical classes; this representation can subsequently be used to determine the chemical class subsumers of each molecule. For a formal definition of syntax and semantics of nonmonotonic existential rules as well as decidability proofs, we refer the interested reader to the relevant articles [9,30,31].

For each chemical entity that we model using rules, we also provide its axiomatisation in the surface syntax—a less-logician-like syntax which we designed and which enables the ontological description of structured objects without the use of logic. Our surface syntax is in the same style of the Manchester OWL syntax [32] and draws inspiration from a syntax suggested for OWL 2 rules [33]. The main motivation for designing this syntax is to provide a means for creating ontological descriptions in a more succinct way and without the use of special symbols. We have formally defined the surface syntax and its translation into nonmonotonic existential rules, but we have not implemented an ontology editor that would allow to write axioms in the new syntax. Similarly, we have not conducted experiments evaluating the use of surface syntax by application experts, but given that the Manchester OWL syntax has been well received by non-logicians [32] and there is active development of tools for supporting more human readable ontology query languages [34], we believe that the suggested syntax has the potential to facilitate curating tasks. Since our main focus is to illustrate the transformation of molecular graphs and chemical class definitions into rules, we omit the technical details and describe our methodology by means of running examples. For a complete specification of the surface syntax including a BNF grammar and mappings to nonmonotonic existential rules we provide an online technical report [35].

#### Molecular structures

**Figure 1:**
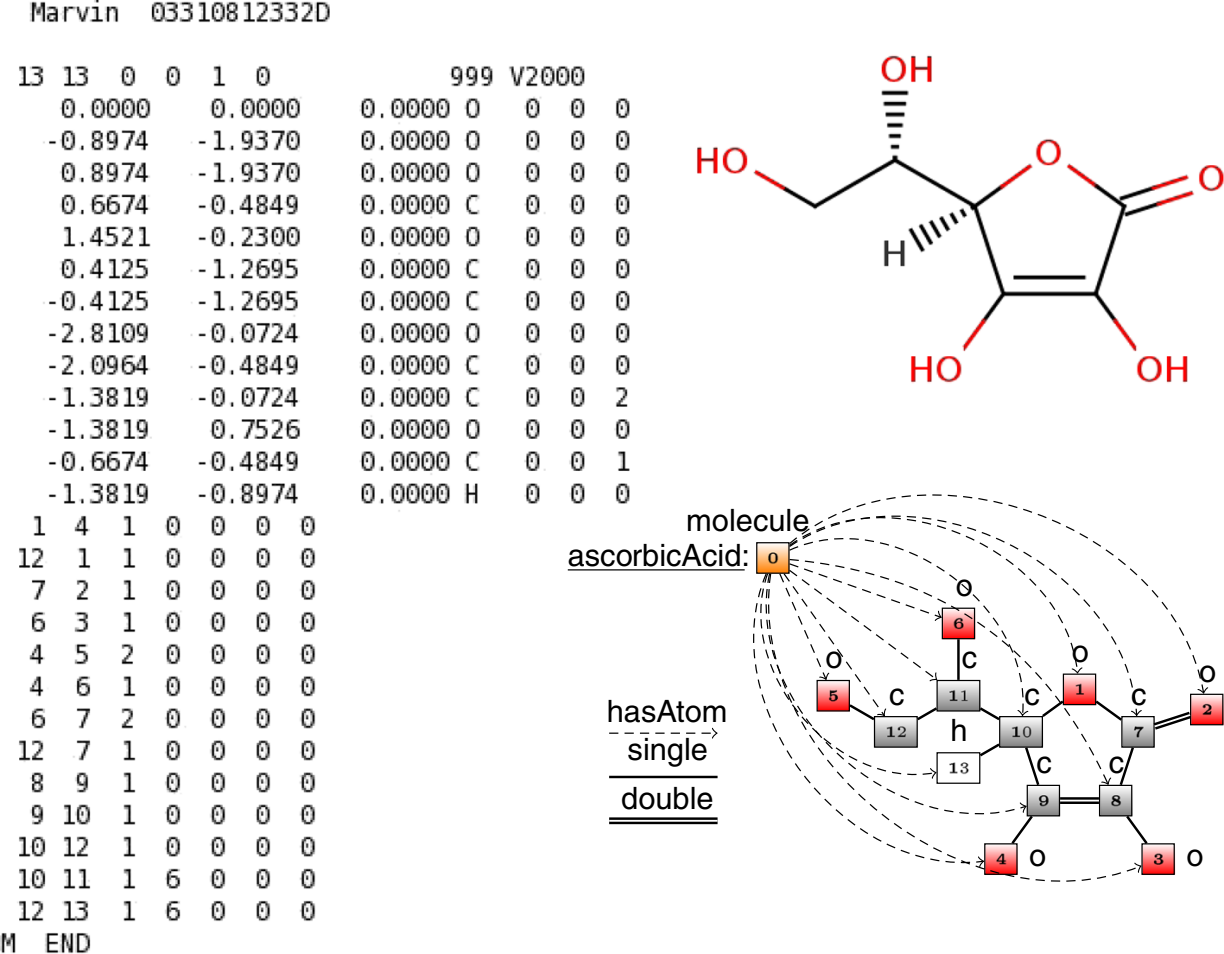
**Ascorbic acid representations.** Molfile **(left)**, molecular graph **(top right)** and description graph (bottom right) encoding the molecular structure of ascorbic acid.

Next, we describe how a molfile can be converted into a surface syntax axiom and subsequently a rule that encodes its structure. We use as an example the molecule of ascorbic acid, a naturally occurring organic compound commonly known as vitamin C. The molecular graph of ascorbic acid is depicted in the upper right corner of Figure 1.

Conceptually, the structure of ascorbic acid can be abstracted with the help of a directed labeled graph such as the one that appears in the lower right corner of Figure 1 and which in our framework is called *description graph* (DG) [9]. The description graph of a molecule is a labeled graph whose nodes correspond to the atoms of the molecule (nodes 1–13 for ascorbic acid) plus an extra node for the molecule itself (node 0) and whose edges correspond to the bonds of the molecule (e.g. (1,7)) plus some additional edges that connect the molecule node with each one of the atom nodes (e.g. (0,1)); additionally, the atom nodes are labeled with the respective chemical elements (e.g. o for node 1) and the bond edges with the corresponding bond order (e.g. single for (1,7)); finally, the molecule node is labeled with molecule and the edges that connect the molecule node with each of the atom nodes are labeled with hasAtom. In order to simplify the depiction of the ascorbic acid DG in Figure 1 a legend is used for the edge labels; all arrowless edges are assumed to be bidirectional. In our setting, we follow the implicit hydrogen assumption according to which hydrogen atoms are usually suppressed (excluding cases where stereochemical information is provided for the formed bond and hydrogens are explicitly stated as in node 13). Finally, we point out that both the nodes and the edges can have multiple labels, allowing us to also encode molecular properties, such as charge values for atoms. The description graph of ascorbic acid can be converted into the following surface syntax definition. In the rest of the text we use alphanumeric strings starting with a lower-case letter to denote predicates, that is names of classes (e.g. ascorbicAcid) and properties (e.g. hasAtom).

$$\begin{array}{ll} \text{ascorbicAcid}\mathrm{SubClassOf}\\ \text{molecule}\;\text{AND}\;(\text{hasAtom}\;\text{SOME}\;\text{Graph}( & \text{Nodes}(\text{1 o},\text{2 o},\text{3 o},\text{4 o},\text{5 o},\text{6 o},\text{7 c},\text{8 c},\text{9 c},\\ & \;\text{10 c},\text{11 c},\text{12 c},\text{13 h)}\\ & \text{Edges}(\text{1 2 single},\text{1 10 single},\text{2 7 double},\text{3 8 single}\\ & \;\text{4 9 single},\text{5 12 single},\text{6 11 single},\text{7 1 single}\\ & \;\text{8 7 single},\text{9 8 double},\text{10 9 single},\text{11 10 single}\\ & \;\text{12 11 single},\text{13 10 single}))) \end{array}$$

The surface syntax axiom above can next be translated into the rule below. In fact we need a separate rule for each conjunct in the head but we use just one rule here to simplify the presentation; for the sake of brevity only one direction of the bonds appear and we shorten an expression of the form ∧*C*_1_…∧*C*_*n*_ with $\wedge_{i=1}^{n}C_{i}$:

$$\begin{array}{lll} \mathrm{ascorbicAcid}(x)\to & \text{molecule}(x)\wedge_{i=1}^{13}\mathrm{hasAtom}(x,f_{i}(x))\; & \;\\ & \wedge_{i=1}^{6}o(f_{i}(x))\wedge_{i=7}^{12}c(f_{i}(x))\wedge h(f_{13}(x))\; & \;\\ & \wedge \text{single}(f_{8}(x),f_{3}(x))\wedge \text{single}(f_{9}(x),\; & \;\\ & \;f_{4}(x))\wedge_{i=1,9,11,13}\mathrm{single}(f_{10}(x),f_{i}(x))\; & \;\\ & \wedge_{i=5,11}\mathrm{single}(f_{12}(x),f_{i}(x))\wedge_{i=1,8}\; & \;\\ & \;\mathrm{single}(f_{7}(x),f_{i}(x))\wedge \text{single}(f_{11}(x),\; & \;\\ & \;f_{6}(x))\wedge \text{double}(f_{2}(x),f_{7}(x))\wedge \; & \;\\ & \;\text{double}(f_{8}(x),f_{9}(x))\; & \; \end{array}$$

The rule above is a typical first-order implication with a single atomic formula in the body and a conjunction of atomic formulae in the head. Informally, the rule ensures that every time that the ascorbic acid molecule instantiated, its structure is unfolded according to its specified DG. Thus, triggering of the rule implies that (i) new terms that correspond to the DG’s nodes are generated (excluding node 0), e.g. *f*_1_(x) represents atom node 1 (ii) each new term is typed according to the label of the relevant node with the help of a unary atomic formula (e.g. o(*f*_1_(x))) and (iii) each pair of terms with corresponding nodes connected in the DG is assigned the respective label with the help of a binary atomic formula (e.g. single(*f*_1_(x),*f*_7_(x))). In order to ensure disjointness of the several molecular structures on the interpretation level, distinct function symbols are used in the rule of each molecule.

#### General chemical knowledge and chemical classes

Before presenting the modelling of various chemical classes, we demonstrate how we can encode background chemical knowledge with surface syntax axioms that can subsequently be mapped to rules. Three such axioms appear next.

$$\begin{array}{l} \text{bond}\;\text{SuperPropertyOf}\\ \text{single}\;\text{OR}\;\text{double}\;\text{OR}\;\text{triple} \end{array}$$

$$\begin{array}{l} \text{charged}\;\text{SuperClassOf}\\ \text{positive}\;\text{OR}\text{negative} \end{array}$$

$$\begin{array}{l} \text{horc}\;\text{SuperClassOf}\\ \text{h}\;\text{OR}\;\text{c} \end{array}$$

Examples of such knowledge include the fact that single and double bonds are kinds of bonds or that atoms with positive or negative charge are charged; we can also denote a particular class of atoms, e.g. atoms that are hydrogens or carbons. The translation of the above mentioned surface syntax axioms into rules appears below.

$$\begin{array}{cccccc} \text{single}(x,y)\to & \text{bond}(x,y) & \text{negative}(x)\to & \text{charged}(x) & h(x)\to & \text{horc}(x)\\ \text{double}(x,y)\to & \text{bond}(x,y) & \text{positive}(x)\to & \text{charged}(x) & c(x)\to & \text{horc}(x)\\ \text{triple}(x,y)\to & \text{bond}(x,y) \end{array}$$

For our experiments, we represented 51 chemical classes using rules; we based our chemical modelling on the textual definitions found in the ChEBI ontology [16].

We covered a diverse range of classes that can be categorised into four groups. For each class that we discuss, we provide the surface syntax definition and its corresponding translation into one or more rules. Certain classes with an intricate definition (such as the class of cyclic molecules that appears later) are not expressible in surface syntax; these can be directly added as rules. Here we show in full detail only a sample of the rules; the complete set of rules is available in Additional files 1, 2 and 3[36].

##### Existence of subcomponents

The great majority of the modelled chemical classes is defined via containment of atoms, functional groups or other atom arrangements. Examples of this type include carbon molecular entities, halogens, molecules that contain a benzene ring, carboxylic acids, carboxylic esters, polyatomic entities, amines, aldehydes and ketones. Next we show the surface syntax axioms that define the classes of carbon molecular entities, polyatomic entities, carboxylic acids and esters. In the following axioms we use the keyword ‘ GraphNL’ in contrast to the previously used ‘ Graph’ as our surface syntax grammar requires the use of the former when specifying nodes that are either labeled with negative literals or are specified to be disjoint.

$$\begin{array}{l} \text{carbonEntity}\;\text{SuperClassOf}\\ \text{hasAtom}\;\text{SOME}\;\text{c} \end{array}$$

$$\begin{array}{ll} \text{polyatomicEntity}\;\text{SuperClassOf}\\ \text{molecule}\;\text{AND}\;(\text{hasAtom}\;\text{SOME}\;\text{GraphNL}( & \text{DisjointNodes}(\text{1},\text{2})\\ & \text{Edges}())) \end{array}$$

$$\begin{array}{l} \text{heteroOrganicEntity}\;\text{SuperClassOf}\\ \text{hasAtom}\;\text{SOME}\text{GraphNL}\;(\text{Nodes}\;(\text{1}\text{c},\text{2}\text{NOT}\;\text{c}\;\text{NOT}\;\text{h})\\ \;\text{Edges}\;(\text{1 2}\;\text{bond})) \end{array}$$

$$\begin{array}{ll} \text{middleOxygen}\text{SuperClassOf}\\ \text{o}\;\text{AND}\;(\text{bond}\;\text{SOME GraphNL}( & \text{DisjointNodes}\;(\text{1},\text{2})\\ & \text{Edges}())) \end{array}$$

$$\begin{array}{ll} \text{carboxylicAcid}\text{SuperClassOf}\\ \text{molecule}\;\text{AND}\;(\text{hasAtom}\text{SOME GraphNL} & (\text{Nodes}\;(\text{1 c},\text{2 o},\text{3 o NOT middleOxygen NOT charged},\\ & \;\text{4 horc)}\\ & \text{Edges}\;(\text{1 2 double},\text{1 3 single},\text{1 4 single}))) \end{array}$$

$$\begin{array}{ll} \text{carboxylicEster}\text{SuperClassOf}\\ \text{molecule}\;\text{AND}\;(\text{hasAtom}\text{SOME Graph} & (\text{Nodes}\;(\text{1 c},\text{2 o},\text{3 o},\text{4 c},\text{5 horc)}\\ & \;\text{Edges}\;(\text{1 2 double},\text{1 3 single},\text{1 5 single},\text{3 4 single}))) \end{array}$$

One can find below the corresponding translations into rules. We define as carbon molecular entities the molecules that contain carbon; polyatomic entities are the entities that contain at least two different atoms. Heteroorganic entities are the ones containing carbon atoms bonded to non-carbon atoms. Carboxylic acids are defined as molecules containing at least one carboxy group (a functional group with formula C(=O)OH) attached to a carbon or hydrogen; due to the implicit hydrogens assumption we are not able to distinguish between an oxygen and a hydroxy group and, so, we need to specify that the oxygen of the hydroxy group is not charged (NOTcharged) and participates to only one bond (NOTmiddleOxygen). Similarly, carboxylic esters contain a carbonyl group connected to an oxygen ((C=O)O) which is further attached to two atoms that are carbon or hydrogen.

() $$\begin{array}{lll} \text{molecule}(x)\wedge \text{hasAtom}(x,y)\wedge c(y)\to & \text{carbonEntity}(x)\; & \;\\ \text{molecule}(x)\wedge \text{hasAtom}(x,y_{1})\wedge \text{hasAtom}(x,y_{2})\wedge y_{1}\neq y_{2}\to & \text{polyatomicEntity}(x)\; & \;\\ \wedge_{i=1}^{2}\text{hasAtom}(x,z_{i})\wedge c(z_{1})\wedge \mathrm{not}c(z_{2})\wedge \mathrm{not}h(z_{2})\wedge \text{bond}(z_{1},z_{2})\to & \text{heteroOrganicEntity}(x)\; & \;\\ \wedge_{i=1}^{3}\mathrm{hasAtom}(x,y_{i})\wedge o(y_{1})\wedge_{i=2}^{3}\mathrm{bond}(y_{1},y_{i})\wedge y_{2}\neq y_{3}\to & \text{middleOxygen}(y_{1})\; & \;\\ \;\text{molecule}(x)\wedge_{i=1}^{4}\mathrm{hasAtom}(x,y_{i})\wedge c(y_{1})\wedge o(y_{2})\wedge o(y_{3})\wedge \;\;\;\; & \;\\ \;\text{horc}(y_{4})\wedge \text{double}(y_{1},y_{2})\wedge \text{single}(y_{1},y_{3})\wedge \text{single}(y_{1},y_{4})\wedge \;\;\;\; & \;\\ \mathrm{not}\text{middleOxygen}(y_{3})\wedge \mathrm{not}\text{charged}(y_{3})\to & \text{carboxylicAcid}(x)\; & \;\\ \;\text{molecule}(x)\wedge_{i=1}^{5}\mathrm{hasAtom}(x,y_{i})\wedge_{i=1,4}c(y_{i})\wedge_{i=2,3}o(y_{i})\wedge \;\;\;\;\; & \;\\ \text{horc}(y_{5})\wedge \text{double}(y_{1},y_{2})\wedge_{i=3,5}\mathrm{single}(y_{1},y_{i})\wedge \text{single}(y_{3},y_{4})\to & \text{carboxylicEster}(x)\; & \; \end{array}$$

##### Exact cardinality of parts

Here we describe chemical classes of molecules with an exact number of atoms or of functional groups. Examples include molecules that contain exactly two carbons, molecules that contain only one atom and dicarboxylic acids, that is molecules with exactly two carboxy groups. The surface syntax axiom for the definition of molecules with exactly two carbons appears next.

$$\begin{array}{l} \text{exactly2Carbons}\text{SuperClassOf}\\ \text{molecule}\;\text{AND}\;\text{hasAtom}\;\text{EXACTLY}\;2\;\text{c} \end{array}$$

The translation into rules follows. One can readily verify that the surface syntax formulation is more direct and intuitive than its equivalent translation into rules.

$$\begin{array}{lll} \text{molecule}(x)\wedge_{i=1}^{2}\mathrm{hasAtom}(x,y_{i})\wedge c(y_{i})\wedge y_{1}\neq y_{2}\to & \text{atLeast}2\text{Carbons}(x)\; & \;\\ \text{molecule}(x)\wedge_{i=1}^{3}\mathrm{hasAtom}(x,y_{i})\wedge c(y_{i})\wedge_{i=2}^{3}y_{1}\neq y_{i}\wedge y_{2}\neq y_{3}\to & \mathrm{atLeast}3\text{Carbons}(x)\; & \;\\ \text{atLeast}2\text{Carbons}(x)\wedge \;\;\mathrm{not}\;\;\text{atLeast}3\text{Carbons}(x)\to & \text{exactly}2\text{Carbons}(x)\; & \; \end{array}$$

##### Exclusive composition

We next present classes of molecules such that each atom (or bond) they contain satisfies a particular property. These features are usually very naturally modelled with the help of nonmonotonic negation. Examples include inorganic molecules that consist exclusively of non-carbon atoms. In spite of the fact that there are many compounds with carbons considered inorganic, in this work we align our encoding with the ChEBI definition of inorganic molecular entities (CHEBI:24835), according to which no carbons occur in these entities; however, if the modeller wishes it, it is straightforward to declare exceptions within our formalism using nonmonotonic negation. Another example is the class of hydrocarbons which only contain hydrogens and carbons; also saturated compounds are defined as the compounds whose carbon to carbon bonds are all single. The corresponding surface syntax axioms appear next.

$$\begin{array}{l} \text{inorganic}\text{SuperClassOf}\\ \text{molecule}\;\text{AND}\;\text{hasAtom}\;\text{ONLY}\;(\;\text{NOT}\;\text{c}) \end{array}$$

$$\begin{array}{l} \text{hydroCarbon}\text{SuperClassOf}\\ \text{carbonEntity}\;\text{AND}\;\text{hasAtom}\;\text{ONLY}\;(\text{h}\;\text{OR}\;\text{c}) \end{array}$$

$$\begin{array}{ll} \text{unsaturated}\text{SuperClassOf}\\ \text{molecule}\;\text{AND}\;\text{hasAtom}\text{SOME Graph}\;( & \text{Nodes}\;(\text{1 c},\text{2 c)}\\ & \text{Edges}\;(\text{1 2 double})) \end{array}$$

$$\begin{array}{ll} \text{unsaturated}\text{SuperClassOf}\\ \text{molecule}\;\text{AND}\;\text{hasAtom}\text{SOME Graph}( & \text{Nodes}(\text{1 c},\text{2 c})\\ & \text{Edges}\;(\text{1 2 triple})) \end{array}$$

$$\begin{array}{l} \text{saturated}\text{SuperClassOf}\\ \text{molecule}\;\text{AND}\;\text{NOT}\;\text{unsaturated} \end{array}$$

Please note that one can use more than one surface syntax axioms (and thus rules) to define classes that emerge as a result of different structural configurations, which is the case for saturated molecules. Below we list the respective translation into rules.

$$\begin{array}{ll} \text{molecule}(x)\wedge \mathrm{not}\text{carbonEntity}(x) & \to \text{inorganic}(x)\\ \text{hasAtom}(x,z)\wedge \mathrm{not}\text{carbon}(z)\wedge \mathrm{not}\text{hydrogen}(z) & \to \text{notHydroCarbon}(x)\\ \text{carbonEntity}(x)\wedge \mathrm{not}\text{notHydroCarbon}(x) & \to \text{hydroCarbon}(x)\\ \text{molecule}(x)\wedge \text{hasAtom}(x,z_{1})\wedge \text{carbon}(z_{1})\\ \text{hasAtom}(x,z_{2})\wedge \text{carbon}(z_{2})\wedge \text{double}(z_{1},z_{2}) & \to \text{unsaturated}(x)\\ \text{molecule}(x)\wedge \text{hasAtom}(x,z_{1})\wedge \text{carbon}(z_{1})\\ \text{hasAtom}(x,z_{2})\wedge \text{carbon}(z_{2})\wedge \text{triple}(z_{1},z_{2}) & \to \text{unsaturated}(x)\\ \text{molecule}(x)\;\;\wedge \mathrm{not}\;\;\text{unsaturated}(x) & \to \text{saturated}(x) \end{array}$$

##### Cyclicity-related classes

These chemical classes include the category of molecules containing a ring of any length as well as other definitions that depend on the cyclicity of molecules, such as alkanes which are defined as saturated non-cyclic hydrocarbons. Assuming the (somewhat more technical) definition of cyclic molecules, the surface syntax axiom for alkanes appears next.

$$\begin{array}{l} \text{alkane}\text{SuperClassOf}\\ \text{saturated}\;\text{AND}\;\text{hydroCarbon}\;\text{AND}\;\text{NOT}\;\text{cyclic} \end{array}$$

The corresponding rule translation follows.

$$\begin{array}{ll} \text{saturated}(x)\wedge \text{hydroCarbon}(x)\wedge \mathrm{not}\text{cyclic}(x)\to \text{alkane}(x)\; & \; \end{array}$$

#### Determining subclass relations

Finally, we demonstrate how meaningful subsumptions can be derived using a KB containing the rules outlined in the previous two sections. In order to determine the superclasses of a certain molecule, we extend the KB with a suitable fact (i.e., a variable-free atomic formula) and we examine the model that satisfies the KB under the *stable model semantics* (the addition of the fact and the examination of the model is done automatically by our implementation). A formal definition of the stable model semantics is provided by Gelfond and Lifschitz [37]. Intuitively, the stable model of a KB is the minimal set of facts that are derived by exhaustively applying the existing rules under a particular rule order; a rule is applied if its positive body can be matched to the so far derived facts and no atom of the negative body is in the already produced set of facts for the said matching.

The initially added fact is the molecule name predicate instantiated with a fresh constant so that the rule that encodes the structure of that molecule is triggered. For the case of ascorbic acid, if we append the fact ascorbicAcid(a) to the previously described KB, we obtain the stable model that appears below.

$$\begin{array}{l} \text{Stable model for ascorbic acid}\\ \text{Input fact:}\text{ascorbicAcid}(a)\\ \text{Stable model:}\;\mathrm{ascorbicAcid}(a),\;\text{molecule}\left(a\right),\;\text{hasAtom}\;\left(a,a_{i}^{f}\right)\;\;\text{for}\;\;1\leq i\leq 13,\;o\;\left(a_{i}^{f}\right)\;\;\text{for}\;\;1\leq i\leq 6,\\ c\;\left(a_{i}^{f}\right)\;\;\text{for}\;\;7\leq i\leq 12,\;h\;\left(a_{13}^{f}\right),\;\text{single}\;\left(a_{8}^{f},a_{3}^{f}\right),\;\text{single}\;\left(a_{9}^{f},a_{4}^{f}\right),\;\text{single}\;\left(a_{12}^{f},a_{i}^{f}\right)\;\;\text{for}\;\;i\in \{5,11\},\\ \mathrm{single}\;\left(a_{10}^{f},a_{i}^{f}\right)\;\;\text{for}\;\;i\in \{1,9,11,13\},\;\text{single}\;\left(a_{7}^{f},a_{i}^{f}\right)\;\;\text{for}\;\;i\in \{1,8\},\;\text{single}\;\left(a_{11}^{f},a_{6}^{f}\right),\mathrm{double}\;\left(a_{2}^{f},a_{7}^{f}\right),\\ \text{double}\;\left(a_{8}^{f},a_{9}^{f}\right),\;\text{bond}\;\left(a_{8}^{f},a_{3}^{f}\right),\;\text{bond}\;\left(a_{9}^{f},a_{4}^{f}\right),\;\text{bond}\;\left(a_{12}^{f},a_{i}^{f}\right)\;\;\text{for}\;\;i\in \{5,11\},\;\text{bond}\;\left(a_{11}^{f},a_{6}^{f}\right),\\ \mathrm{bond}\;\left(a_{10}^{f},a_{i}^{f}\right)\;\;\text{for}\;\;i\in \{1,9,11,13\},\;\text{bond}\;\left(a_{7}^{f},a_{i}^{f}\right)\;\;\text{for}\;\;i\in \{1,8\},\mathrm{bond}\;\left(a_{2}^{f},a_{7}^{f}\right),\;\text{bond}\;\left(a_{8}^{f},a_{9}^{f}\right),\\ \text{horc}\;\left(a_{i}^{f}\right)\;\;\text{for}\;\;7\leq i\leq 13,\;\text{carbonEntity}\left(a\right),\;\text{polyatomicEntity}\left(a\right),\;\text{heteroOrganicEntity}\left(a\right),\\ \mathrm{middleOxygen}\;\left(a_{1}^{f}\right),\;\text{carboxylicEster}(a),\;\text{atLeast}2\text{Carbons}\left(a\right),\;\text{atLeast}3\text{Carbons}\left(a\right),\\ \mathrm{notHydroCarbon}\left(a\right),\;\text{unsaturated}\left(a\right),\;\text{cyclic}\left(a\right)\\ \text{Stable model of the KB with the input fact}\mathrm{ascorbicAcid}(a)\text{and the rules described in Methods;}f_{i}(a)\text{is}\\ \text{abbreviated with}a_{i}^{f}\;\;\text{for}\;\;1\leq i\leq 13. \end{array}$$

From the stable model atoms we can infer the superclasses of ascorbic acid, that is we deduce that ascorbic acid is—among others—an unsaturated, polyatomic, heteroorganic, cyclic molecular entity that contains carbon and a carboxylic ester. If there is no relevant atom for a chemical class in the stable model, then we conclude that the said class is not a valid subsumer, e.g. since carboxylicAcid(a) is not found in the stable model, carboxylic acid is not a superclass of ascorbic acid.

#### Decidability check

The KB discussed above contains rules with function symbols in the head, such as the rule used to encode the molecular structure of ascorbic acid. These rules may incur non-termination during the computation of the stable model due to the creation of infinitely many terms. In order to ensure termination of our reasoning process and thus decidability of the employed formalism, we perform a *decidability* check on the constructed KB. In a nutshell, the decidability check (also known and as *model-summarising acyclicity*[38]) involves transforming the rules of the KB and inspecting the stable models of the transformed KB for the existence of a special symbol. If the KB passes the decidability check, then termination is guaranteed; this is the case for the types of KBs that were previously described. Technical details of the aforementioned condition are out of the scope of this text and can be found in the relevant sources [38].

### Prototype implementation

**Figure 2:**
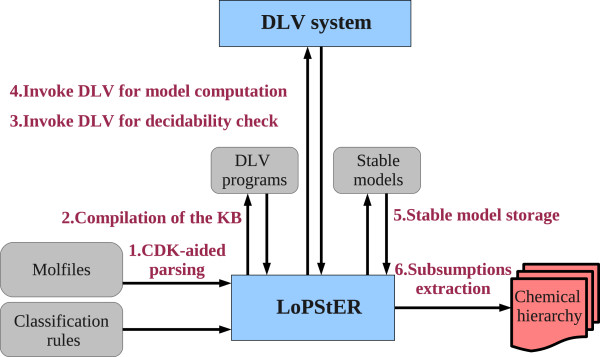
**Architecture of LoPStER.** Stages of the classification process using LoPStER.

The current section provides an overview of LoPStER (**Lo**gic **P**rogramming for **St**ructured **E**ntities **R**easoner) the prototype we developed for structure-based chemical classification. The implementation is wrapped around the DLV system, a powerful and efficient deductive database and logic programming engine [39]. DLV constitutes the automated reasoning component used by LoPStER for stable model computation of a rule set. Figure 2 depicts the basic processing steps as well as the different files that are parsed and produced by LoPStER. LoPStER is implemented in Java and is available online [36]; both LoPStER and the rules modelling chemical classes are open-source and released under GNU Lesser GPL. Next, we describe in more detail the several stages of execution. 

1. **CDK-aided parsing.** LoPStER parses the molfiles [40] of the molecules to be classified using the Chemistry Development Kit Java library [41]. The molfile is a widely used chemical file format that describes molecular structures with a connection table; e.g. the molfile of ascorbic acid appears on the left of Figure 1. For each molecule, a description graph (e.g. Figure 1 bottom right) representation is generated from its molfile according to a transformation as the one described for ascorbic acid.

2. **Compilation of the KB.** For each molecule the description graph representation is used to produce a set of rules that encode the structure of the molecule, following the translation that was discussed in the previous section. These rules along with the classification rules and the facts necessary to determine subclass relations are combined to produce DLV programs (i.e. sets of rules) that are stored as plain text files on disk. In particular two kinds of DLV programs are created for each molecule, the program needed to perform the decidability check as described before and the program needed to compute subclass relations between the molecules and the chemical classes.

3. **Invoke DLV for decidability check.** During this step, the model of the program, which was produced in the previous step for acyclicity testing, is computed. If the check is successful, then execution proceeds to the next stage; otherwise, the program is exited with a suitable output message.

4. **Invoke DLV for model computation.** This is the stage where DLV is invoked to compute the stable model of the KB. Due to the check of the previous step, the computation is guaranteed to terminate.

5. **Stable model storage.** At this point, the stable model computed by DLV is stored in a file on disk to enable subsequent discovery of the subclass relations.

6. **Subsumptions extraction.** This is the final phase where the stable model file is parsed in order to detect the superclasses of each molecule. All the subsumee-subsumer pairs are stored in a separate spreadsheet file on disk.

## Results

### Empirical evaluation

In order to assess the applicability of our implementation, we measured the time required by LoPStER to perform classification of molecules. To obtain test data we extracted molfile descriptions of 500 molecules from the ChEBI ontology. The represented compounds were of diverse size, varying from 1 to 59 atoms. Next, we investigated the scalability of our prototype by altering two different parameters of the knowledge base, namely the number of represented molecules and the type of modelled chemical classes. Initially, we constructed ten DLV programs each of which contained rules encoding 50·*i* different compounds, where 1≤*i*≤10, and rules defining the chemical classes (a sample of which was previously described) excluding the cyclicity-related classes (48 classes in total). Next, we repeated the same construction but this time including the rules for the cyclicity-related classes (51 classes in total). In the rest of the section, we refer to the first setting as ‘no cyclic’ and to the second as ‘with cyclic’.

Additionally and in order to optimise the performance, we explored how classification times fluctuate depending on the size of DLV programs. In particular, we partitioned the DLV programs into modules, we measured classification times for each module separately and we summed up the times. Each module contains the facts and the rules describing a subset of the molecules represented in the initial DLV program; the rules defining chemical classes are included in each one of the modules. Thus, the size of each module depends on the number of encoded molecules. We tested modules of various sizes as well as DLV programs without any partitioning for both ‘no cyclic’ mode and ‘with cyclic’ mode. Modifying the size of the module had a clear impact on the measured times and performing classification with the modularised knowledge base was always quicker than with the unpartitioned one; we observed the shortest execution times for module size 50 when testing in ‘no cyclic’ mode and for module size 20 when testing in ‘with cyclic’ mode; the timings we provide next refer to the aforementioned module sizes.

**Figure 3:**
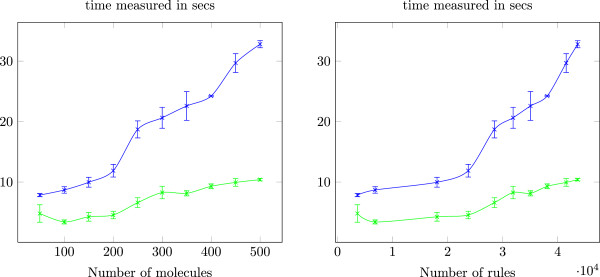
**Classification times.** Curves of classification times with respect to number of molecules **(left)** and number of rules **(right)**. The lower line is for ‘with cyclic’ mode and the upper for ‘no cyclic’ mode.

Table 1 summarises the classification times for the previously described KBs. All the DLV programs that were tested passed the decidability check. The experiments were performed on a desktop computer (2GHz quadcore CPU, 4GB RAM) running Linux. The first column displays the number of molecules, the second column the number of rules contained in the corresponding DLV program and the third (fourth) column the time needed to perform classification in ‘no cyclic’ (‘with cyclic’) mode. We only display the number of rules for the ‘no cyclic’ mode because there are only six more rules in the DLV programs with cyclicity-related definitions. The classification experiments for each knowledge base were repeated three times and the results were averaged over the three runs; also, the durations of Table 1 are inclusive, that is they count the time spent from before the molfiles parsing until after the subsumptions extraction. Figure 3 depicts the plots of the time intervals appearing in Table 1 both with regard to the number of molecules and the number of rules contained in the respective DLV program.

**Table 1:** Time measurements for classification

**No molecules**	**No of rules**	**Time no cyclic**	**Time with cyclic**
		**(sec)**	**(sec)**
50	3614	4.81	7.85
100	6832	3.41	8.69
150	18072	4.25	9.97
200	23746	4.55	11.88
250	28502	6.60	18.71
300	31892	8.27	20.63
350	35046	8.14	22.58
400	38095	9.30	24.23
450	41536	9.94	29.68
500	43629	10.40	32.79

The first column is the number of molecules, the second is the number of rules in the corresponding rule set and the third and fourth are measurements in seconds for ‘no cyclic’ and ‘with cyclic’ mode, respectively.

The performance results of Table 1 are encouraging for the practical feasibility of our approach: the classification of 500 molecules was completed in less than 33 secons for the suite of 51 modelled chemical classes. The drop in classification times between the 50 and 100 molecules case is potentially due to JVM startup overhead. One can also observe that the rules encoding cyclicity-related classes introduce a significant overhead for the classification times. In fact, it is the class that recognises molecules with cycles of arbitrary length that incurs the performance penalty. The rules that encode the class of cyclic molecules need to identify patterns that are extremely frequent in molecular graphs; as a consequence, the amount of computational resources needed to detect ring-containing molecules is much higher. However, since our class definition for cyclic molecules detects compounds with cycles of variable length, which is a significant property for the construction of chemical hierarchies, we consider this overhead acceptable.

### Discussion and related work

Concerning expressive power, the current approach allows for the representation of strictly more chemical classes in comparison with other logic-based applications for chemical classification. Villanueva-Rosales and Dumontier [19] describe an OWL ontology of functional groups for the classification of chemical compounds; in their work, they point out the inherent inability of OWL to represent cyclic functional groups and how this impedes the use of OWL in logic-based chemical classification. As a remedy, Hastings et al. [21] employ an extension of OWL [42] for the representation of non-tree-like structures and, thus, for the classification of molecular structures. However, the used formalism only allows for the identification of cycles of fixed length and with alternating single and double bonds. In the current approach we are able to recognise molecules containing cycles of both arbitrary and fixed length and without requiring a particular configuration of bonds.

Moreover, in both approaches outlined above the adopted open world assumption of OWL prevents one from defining structures based on the absence of certain characteristics. In our approach we operate under the closed world assumption which permits the definition of a broad range of chemical classes that were not expressible before such as the class of inorganic, hydrocarbon or saturated compounds. Finally and in comparison with previous work [9], we take full advantage of the suggested formalism by specifying a much wider range of chemical classes and we do not require from the modeller a precedence relation between the represented structures.

In terms of performance, the classification results appear more promising than previous and related work. Hastings et al. [21] report that a total of 4 hours was required to determine the superclasses of 140 molecules, whereas LoPStER identifies the chemical classes of 500 molecules in less than 33 seconds. LoPStER is quicker in comparison with previous work too [9] where 450 seconds were needed to classify 70 molecules (two orders of magnitude faster). Please note that both cases discussed above considered a subset of the chemical classes used here. Regarding the significant change in speed, we identify the following two factors that could explain it. First, DLV is a more suitable reasoner for our setting due to its bottom-up computation strategy as well as its active maintenance team and frequent releases. Second, we employ a more efficient condition (model-summarising acyclicity [38] instead of semantic acyclicity [9]) in order to obtain termination guarantees which allows for a more prompt decidability check. Finally, the classification times reported here are slightly improved in comparison with a preliminary version of this paper due to some modelling optimisations and the use of a recent new version of DLV.

**Figure 4:**
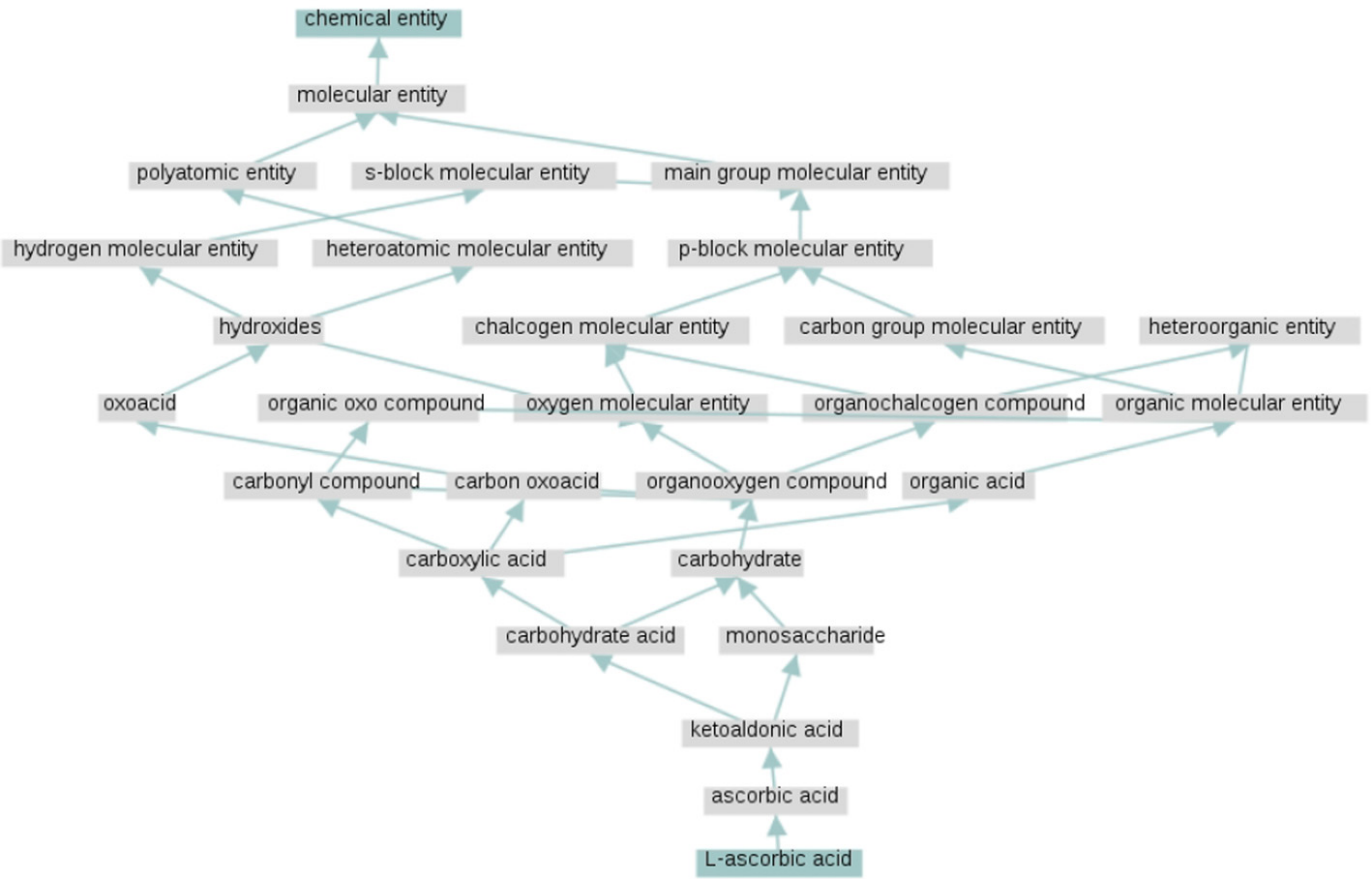
**Ascorbic acid superclasses.** Superclasses of ascorbic acid for the ChEBI OWL ontology release 102 as illustrated by the ChEBI graph-based visualisation interface.

While conducting the experiments we discovered a number of missing and inconsistent subsumptions from the manually curated ChEBI ontology; here we only mention a few of them. As one can infer from the molecular graph of ascorbic acid appearing in the top right of Figure 1, ascorbic acid is a carboxylic ester as well as a polyatomic cyclic entity. In spite of the fact that these superclasses were exposed by our classification methodology, we were not able to identify them in the ChEBI hierarchy. Figure 4 shows the ancestry of ascorbic acid (CHEBI:29073) in the OWL version of the ChEBI ontology; none of the concepts cyclic entity (CHEBI:33595), polyatomic entity (CHEBI:36357) or carboxylic ester (CHEBI:33308) is encountered among the superclasses of ascorbic acid. Moreover, ascorbic acid is asserted as a carboxylic acid (CHEBI:33575) which is not the case as it can be deduced by the lack of a carboxy group in the molecular graph of ascorbic acid (the most common tautomer of which appears in the top right corner of Figure 1). We interpret the revealing of these modelling errors as an indication of the practical relevance of our contribution.

The chemical classification methodology that we present here is similar to other classification efforts based on semantic technologies, such as classification of proteins [7] or lipids [8]. Wolstencroft et al. use a bioinformatics tool to extract composition information from protein descriptions and subsequently translate this information into OWL axioms; these axioms are next used to classify the proteins using a DL reasoner. Chepelev et al. use a cheminformatics tool to process lipid descriptions and produce annotated lipid specifications that are then classified using an OWL ontology. The motivation of these two investigations is similar to ours, i.e. alleviation of biocurating tasks; what distinguishes the two approaches from ours is the use of a different ontology language and the role that this language plays during classification. In particular, in our work we use nonmonotonic existential rules instead of OWL which, unlike OWL, are able to capture cyclic structures. Also, in the sequence of steps followed by our classification process we do not rely on a cheminformatics functionality to algorithmically annotate the molecular descriptions, but instead the identification of structural features forms integral part of reasoning. The framework we suggested can be suitable for the domains of lipids and proteins, as long as they are restricted to structures of finite size; however empirical evaluation would be needed to assess the suitability of the framework in practice. Regarding the application of our prototype to ChEBI classification, it could be used to classify ChEBI molecules under the chemical classes defined here, but more curating effort would be needed to model the thousands of chemical classes that appear in ChEBI.

In this work, we represent and reason about chemical knowledge using an ontology language. However, the majority of axioms constituting the ontology, that is the molecule descriptions, are sourced through molfiles that are parsed using cheminformatics libraries. The information provided by these files includes connectivity between atoms, types of atoms and bonds and charges of atoms. This information is converted into logical axioms that are subsequently processed by an automated reasoning algorithm to identify the chemical classes of the molecules. This approach has the advantage of allowing the knowledge modeller to define new classes in a declarative way, that is without the need of writing code for detecting their subsumees. However, a feature that could be detected using cheminformatics algorithms and become part of the ontology axioms is the existence of ring atoms. The benefits of such a modification could be twofold: it could considerably speed up the computation of all cyclicity-related classes (e.g. determining whether an atom is a ring atom can be done very quickly using the CDK library) and at the same time could allow for the definition of strictly more cyclicity-related classes, such as carbocyclic compounds.

An alternative approach could be to build rules from chemical identifiers other than molfiles, such as InChi [43] or preferred IUPAC names [44]. In particular, InChi with its abilitiy to encode isotopical and stereochemical information (which can be critical for biological applications) could lead to richer chemical modelling. Also, widely used chemical databases, such as ChemSpider [45], could be used as a resource for adding to rules information about molecular properties.

A category of molecules that our framework does not cover is tautomers. A tautomer is each of two or more isomers that exist together in equilibrium, and are readily interchanged by migration of an atom (usually hydrogen) or group within the molecule. InChi handles tautomerism by allowing a compound to contain mobile hydrogen atoms, that is some hydrogens are marked as being able to occur in different positions. This is an approach that could be adopted by our methodology too, if we extended our formalism with the ability to represent disjunctive information. However, enriching nonmonotonic existential rules with disjunction would require to alter the design and implementation of the reasoning algorithm, so treating tautomers could be part of a future extension of our framework.

## Conclusion

We presented an implementation that performs logic-based classification of chemicals and builds upon a sound and complete reasoning procedure for nonmonotonic existential rules; our prototype relies on the DLV system and is considerably quicker than previous approaches. For our evaluation, we represented a wide variety of chemical classes that are not expressible with OWL-based formalisms and described a surface syntax that could enable cheminformaticians to define ontological descriptions of chemical entities intuitively and without the need to use first-order logic notation; additionally, our software revealed subclass relations that are missing from the manually curated ChEBI ontology as well as some erroneous ones. We demonstrated thus the capabilities of a datalog-based ontology language that displays a favourable trade-off between expressive power and performance for the purpose of structure-based classification.

### Future research

**Figure 5:**
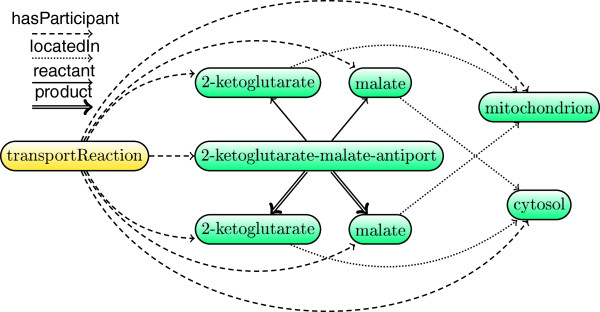
Transport reaction description graph.

**Figure 6:**
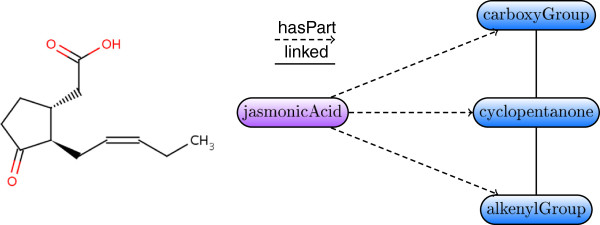
**Jasmonic acid description graph.** Molecular graph of jasmonic acid **(left)** and description graph of jasmonic acid based on the functional groups partonomy **(right)**.

For the future it would be interesting to further apply our framework towards supporting classification of other complex biological objects. For instance, one can exploit the expressive power of rules to represent biochemical processes and infer useful relations about them. Figure 5 depicts a description graph abstraction of a chemical reaction example discussed by Bölling et al. [46]. The process consists of parts that are arbitrarily interconnected and can thus be naturally modelled using our formalism. In the same vein, our methodology could provide rigorous definitions for the representation of lipid molecules that can be systematically classified according to their structural features. Low et al. [47,48] introduced the OWL DL Lipid Ontology which contains semantically explicit lipid descriptions. One could achieve more accurate modelling by casting lipids in terms of rules that capture frequent cyclic patterns in a concise way; for example, Figure 6 illustrates a description graph for jasmonic acid—one of the lipids encountered in the abovementioned OWL ontology.

Further work could involve the building of an ontology editor for the creation of surface syntax expressions and their automatic conversion into nonmonotonic existential rules. We will also seek to extend our prototype to accommodate subsumption between chemical classes so as to generate a complete multi-level chemical hierarchy using ideas from our recent work [49,50]. We could extend our formalism with numerical value restrictions [51] in order to express e.g. classes depending on molecular weight. Moreover, it could be of interest exploring the integration of our prototype with Protégé [52], Life Sciences platforms [53] and chemical structure visualisation tools [54,55] as well as defining a mapping of the introduced formalism to RDF [56].

## Abbreviations

OWL: Web ontology language; ChEBI: Chemical entities of biological interest; W3C: World wide web consortium, KR:Knowledge representation; ML: Machine learning; KB: Knowledge base; DG: Description graph; LoPStER: Logic programming for structure entities reasoner; RDF: RDFResource description framework.

## Competing interests

The authors declare that they have no competing interests.

## Authors’ contributions

All authors conducted research on the underlying decidability conditions for datalog-based rules and jointly discussed the present paper and its main contributions (surface syntax, chemical modelling, experimental setup). DM has specified the surface syntax grammar, assembled the knowledge base, carried out the experiments and led the writing of the manuscript. MK and IH contributed to the discussions and participated in the writing of the manuscript. All authors read and approved the final manuscript.

## Supplementary Material

Additional file 1 **Time measurements and produced hierarchy of the classification experiments.** Description of data: Full list of computed subsumptions and time measurements for each of the five experiments discussed in Empirical evaluation. Click here for file

Additional file 2 **Logic program without cyclicity-related rules.** Description of data: Set of rules modelling the chemical classes excluding the cyclicity-related classes. Click here for file

Additional file 3 **Complete logic program.** Description of data: Set of rules modelling all the chemical classes. Click here for file
